# The societal role of lifelong vaccination

**DOI:** 10.3402/jmahp.v3.26962

**Published:** 2015-08-12

**Authors:** Maarten J. Postma, Stuart Carroll, Alexandra Brandão

**Affiliations:** 1 Unit of Pharmaco Epidemiology & Pharmaco Economics, Department of Pharmacy, University of Groningen, Groningen, Netherlands; 2 Institute of Science in Healthy Aging & Healthcare, University Medical Center Groningen, Groningen, Netherlands; 3 Sanofi Pasteur MSD, Maidenhead, United Kingdom; 4 Sanofi Pasteur MSD, Amadora, Portugal

**Keywords:** vaccination, quality of life, societal, indirect protection, productivity

## Abstract

The full economic and societal value of vaccination is complex to assess. Although direct protection is the immediate goal of vaccination programmes, it is rare that 100% uptake is attained. An important facet of vaccines value comes from the indirect (or herd) protection they provide. The evolving dynamics of our society, including the increase in the proportion of older individuals enhances the value of indirect protection in reducing disease transmission within the family setting and the society as a whole. For example, grandparents are increasingly involved in childcare, putting them at risk of disease transmission if they or the children are not vaccinated. Preventing disease in children can also reduce absenteeism for parents who otherwise would take days off work to care for their sick children, leading to a substantial societal burden. Preventing disease in working adults reduces absenteeism and presenteeism, enhancing productivity and contributing in turn to economic growth. Quality of life is essential at all ages. It is fundamental in children for their life chances, educational achievements, and healthy wellbeing. Additionally, preventing common diseases in adults and the elderly also contributes to their quality of life and helps to assure healthy ageing for growing ageing populations. These wider economic and societal values, although difficult to measure, should be taken into consideration in assessments of the economic value and cost-effectiveness of vaccination programmes.

Vaccination is often regarded as an individual intervention with a wider public health impact. By vaccinating one person, protection can be conferred to a wider group of people through the phenomenon of ‘herd effect’ that provides indirect protection. In today's highly globalised world, the demographic changes occurring and the increasing cross-border population movements and migration between countries and continents have important implications for global health. Controlling the spread of diseases, and related transmission dynamics, is a key challenge in global health, and the role and value of vaccination in this area are obvious. Vaccines have an important role for both preventing disease and reducing societal burden through the prevention of indirect costs of disease, such as absenteeism from work, productivity losses, and working days lost for parents and caregivers. In this article, we will present some examples of vaccination's indirect protection and its associated benefits within the family and the broader society, as well as examples of vaccination's impact on absenteeism, productivity and quality of life, which are of critical importance in the growing elderly populations.

## Enhancing value of vaccination through indirect protection

One of the unique properties of vaccination – and sometimes even potentially one of the main goals – is its ability to confer indirect protection through a herd effect to unvaccinated individuals and groups (1). Vaccination of a fraction of a population reduces the number of those susceptible to infection within the population and, thus, the probability of infection that can result in disease is also reduced. Hence, vaccination can control the transmission of the causative agent and limit the associated infection both directly and indirectly. This benefit is increasingly important given today's globalisation and nomadic cross-border population movements (2) and emphasises the broader value of vaccination as an essential preventative healthcare intervention.

The conjugate pneumococcal vaccine provides an example of the economic benefits of indirect protection. In Germany, the results from a cost-effectiveness study showed that the total costs from pneumoccal disease for the entire German birth cohort were €808.3 million without vaccination and €928.1 million with vaccination, when the benefits from herd effects were not considered (3). The incremental cost per life-year (LY) gained with vaccination was estimated to be over €100,000, that is, likely not cost-effective. However, when herd effect benefits were considered for the same German birth cohort, the total costs were €1,281.4 million with no vaccination and €1,288 million with vaccination. The incremental cost per LY gained with vaccination was below €200/LY gained, that is, highly cost-effective, almost cost-saving. Similar analyses have been performed in other countries, for example, the Netherlands (4). Excluding herd effects in economics evaluations of vaccines thus underestimates the value of vaccination by limiting the concept of value to direct effects only; this can lead to sub-optimal and inefficient public health decisions.

## The value of indirect protection within families and society

Vaccination protects and influences circumstances in whole families. Indirect protection is key if the impact that infectious diseases in children can have on families’ dynamics and caregivers’ absenteeism is considered. For example, a child with varicella may be excluded from school for up to 2 weeks, which corresponds to the disease's incubation period. When the child recovers and returns to school, infected siblings may exhibit symptomatic disease and be off school for another 2 weeks, prolonging parental or caregivers’ absenteeism. The importance of the role of grandparents is also highlighted by policies implemented in some European countries. In Germany, parents are entitled to take leave for up to 3 years after their child is born, 12 months of which can be deferred until the child is eight. Working grandparents may also take 10 days leave in an emergency to care for their grandchildren or to take unpaid leave of up to 6 months (5). In the UK, results from the Millennium Cohort Study showed that in 42% of families, 9-month-old children were looked after by grandparents when parents were at work, illustrating the importance of healthy grandparents also from this viewpoint (6). Changes in parental working patterns, linked with the current economic climate, have reinforced this trend. Therefore, indirect protection has become more important, due to these increasing contacts between these generations. Vaccination of children can reduce transmission to susceptible parents, and particularly older grandparents who can be more susceptible to infectious diseases (i.e., varicella, rubella, and pneumococcal disease) and have a higher risk of severe complications.

The role of indirect protection is also pertinent for wider protection across society, extending protection benefits to population groups that are not, cannot be or will only be reluctantly vaccinated (i.e., newborn infants, pregnant women, immunocompromised individuals). This protection may be crucial in public places, such as public transports, schools, and workplaces. The dynamic nature of mixing and contact patterns within a given population is a strong argument in favour of vaccination to provide both aggregate direct, and subsequent indirect protection to reduce disease transmission within the society as a whole. Even if some of the broader benefits of indirect protection may be difficult to quantify in monetary terms and, thus, challenging to include in economic evaluations (7), public health workers across European countries need to recognise and evaluate these societal benefits to inform policy decisions concerning vaccination and competing alternatives.

## Reducing the societal and caregiver burden

It is increasingly acknowledged that the costs of disease not only fall on the individual patient but also on caregivers including family, friends, communities, and the wider society. It is from this perspective that vaccination against preventable infectious diseases warrants a broader societal value. As mentioned previously, varicella disease in children incurs considerable indirect costs as a result of parental absenteeism and loss of productivity (8, 9). For example, the total indirect costs of varicella over a 50-year period in Italy were estimated to be €2,280 million (10). In Germany, the total annual costs of varicella for payers was estimated to be €78 million, the largest portion of which was due to the significant work loss costs incurred by parents caring for their sick children. For the society, the total annual costs were estimated to be €187.5 million, 82% of which corresponded to indirect costs (11).

Another study conducted in seven European Union (EU) countries estimated the percentage of rotavirus gastroenteritis (RVGE) cases that required at least one parent or another person to be absent from work was up to 91% for hospitalised children, between 4 and 64% for those attending emergency departments and between 20 and 64% for those seen in primary care (12). Similarly, a review conducted in Western Europe showed that between 11 and 61% of parents of children with influenza took leave from work for their own influenza infection or to take care of their children for a mean period of between 1.3 and 6.3 days (13). Bearing the average daily salary in EU countries in mind [median gross hourly earnings of €12 in EU-27, up to €25 in Denmark in 2010 (14)], the example of absenteeism generated by rotavirus and influenza infections equates to a significant indirect costs. In France, the economic burden of productivity loss represents almost 50% of the total cost of RVGE (15). Rotavirus vaccines have demonstrated a high efficacy in reducing the number of work days lost for parents to take care for their children and could thereby reduce this societal burden (16). Lastly, other additional productivity losses and costs associated with replacing staff, social security, or other health insurance payments should be considered. These aspects obviously further strengthen the economic value of vaccination in reducing indirect costs from a societal perspective.

## Minimising workforce absenteeism and improving economic productivity

Absenteeism can have a profound economical impact by undermining productivity in the workplace. For example, in the UK, each absent employee cost their employer an average of £975 in 2012, while absenteeism direct costs alone amounted to more than £14 billion a year across the economy (17). Loss of productivity is a key cost associated with absenteeism followed by the cost of payments for sickness leave and the cost of replacing staff to cover the absent employee. In addition, reduced productivity in the form of presenteeism is a consequence of illness at work and may potentially even outweigh the cost of absenteeism. For example, the total cost of presenteeism to the Australian economy was estimated to be AU$34.1 billion in 2009–2010, equating to a 2.7% decrease in the 2010 gross domestic product (18). The total cost of presenteeism to US employers ranges from about $150 to $250 billion annually, representing about 60% of the total cost of workers’ illness (19). Healthier people can not only work longer, they are also able to work more productively.

As an adjacent issue, vaccination of healthcare workers can help to improve the productivity of healthcare systems, where the level of absenteeism is becoming increasingly problematic, affecting the quality of care and resource management. This is especially pertinent in the case of influenza vaccination. Research suggests that even a 1% decrease in absenteeism of healthcare workers could lead to savings of around £34.2 million for the National Health Service in the UK (20). Additionally, in healthcare workers influenza and pertussis vaccination can provide crucial indirect protection to patients being cared for.

As populations grow older and the retirement age is increased to cope with the financial pressure of retirement funds of social security organisations, avoiding preventable disease in the working population and the ‘young’ elderly becomes more important. For example, herpes zoster (HZ) and post-herpetic neuralgia (PHN) have a negative impact on the productive work life of individuals. In a Canadian study, 64% of the employed participants reported missing work and 76% reported decreased effectiveness at work (i.e., presenteeism) due to HZ and PHN, for a mean number of 43 and 46 h, respectively (21). Vaccinating against HZ, pneumococcal disease, or flu could, therefore, help to contribute to healthy ageing, ensuring that people remain active, independent, and continue to be an asset for society. As described in another paper in this supplement, negative effects from absenteeism in working-aged adults go beyond the healthcare setting, affecting many industrial and service sectors (22). Reducing absenteeism from preventable disease and enhancing productivity are, therefore, essential for generating sustainable economic growth and making healthcare systems more affordable. Vaccination can strongly contribute to these societal challenges.

## Lifelong quality of life and healthy ageing

A central role of public health policy is to protect lives by reducing the burden of infectious diseases and preventing premature deaths. Vaccination has successfully contributed to these key goals. In addition, public health policy also aims to improve the quality of life and promote healthy ageing for all citizens.

European governments have recognised the importance of healthy ageing as part of the inevitable demographic changes occurring in many countries (22, 23). As populations age, there is an inevitable increase in individuals with chronic, long-term conditions. Preventing disease to foster healthy ageing is important, not just in terms of contributing to healthcare systems sustainability and affordability but also from the point of view that elderly individuals often constitute the most active group of volunteers in a society and are central to many community-based projects.

Also, in this context, vaccinations against influenza, pneumococcal disease, and HZ can be considered as quality-of-life enhancing interventions. A recent UK study that investigated the clinical presentation and quality-of-life burden of HZ and PHN from individual, clinical, and societal perspectives found that the pervasive nature of PHN pain and associated symptoms placed significant strains on individual and healthcare resources (24–26). The results also showed that the burden of disease extended beyond pain, with patients experiencing symptoms, such as emotional distress and depression, which all contributed to significant productivity losses. Notably, this societal value extends to the community as a whole. Preventing disease, although vitally important from an economic and employment perspective, is potentially even more fundamental to protecting and enhancing social, personal, and family activities.

## Socio-economic status, health, and missed equity

The principle of equity and equal access to maximise populations’ health is a keystone of modern healthcare systems. Absence of equity and equal access can result in significant missed opportunities, inflating social security and healthcare expenses with the well-known societal consequences that invariably occur. This, in turn, strengthens the adagium ‘prevention is better than cure’ as a general value proposition also as a means of reducing health inequalities. For example, a recent study in the UK showed that hospital admissions for all-cause gastroenteritis in children increased with a lower level of socio-economic status (27). The study concluded that the implementation of a rotavirus vaccination programme would help to reduce the burden of RVGE and all-cause gastroenteritis, and in this context, could have an impact on healthcare and social inequalities. Herd protection from vaccination may also play a role in indirectly protecting populations with lower socio-economic conditions, who may be harder to reach and have poorer access to healthcare and vaccination programmes. Finally, for example, HZ's complications and other infections in advanced age may lead to early retirement, impacting on retirement plans and potentially affecting socio-economic conditions of pensionados.

## Conclusions

Vaccination leads to lifelong individual and societal benefits, helping to reduce indirect costs, such as productivity losses and absenteeism from work, and improve quality of life. These key aspects of value contribute to equity and, in turn, avoid unequal access and health differences related to socio-economic status. Evaluations of vaccines should, therefore, consider these wider dimensions of value, particularly in the context of indirect protection. The societal benefits resulting from vaccination, although difficult to ascertain, should not be underestimated; they are fundamental to the true value proposition of vaccination. Wider social value, in addition to economic value, should be captured as part of routine assessment of the economic interest and cost-effectiveness of vaccination programmes.
